# Supplementary material for: Perivascular localization of macrophages in the intestinal mucosa is regulated by Nr4a1 and the microbiome
Source: Nat Commun. 2020 Mar 12;11:1329. doi: 10.1038/s41467-020-15068-4 (PMC7067862; doi:10.1038/s41467-020-15068-4)
Supplement: Supplementary file 1 — Supplementary Figure [file 41467_2020_15068_MOESM1_ESM.pdf]

Perivascular Localization of Macrophages in the Intestinal Mucosa is Regulated by Nr4a1 and the Microbiome

Honda et al.

Supplementary Information

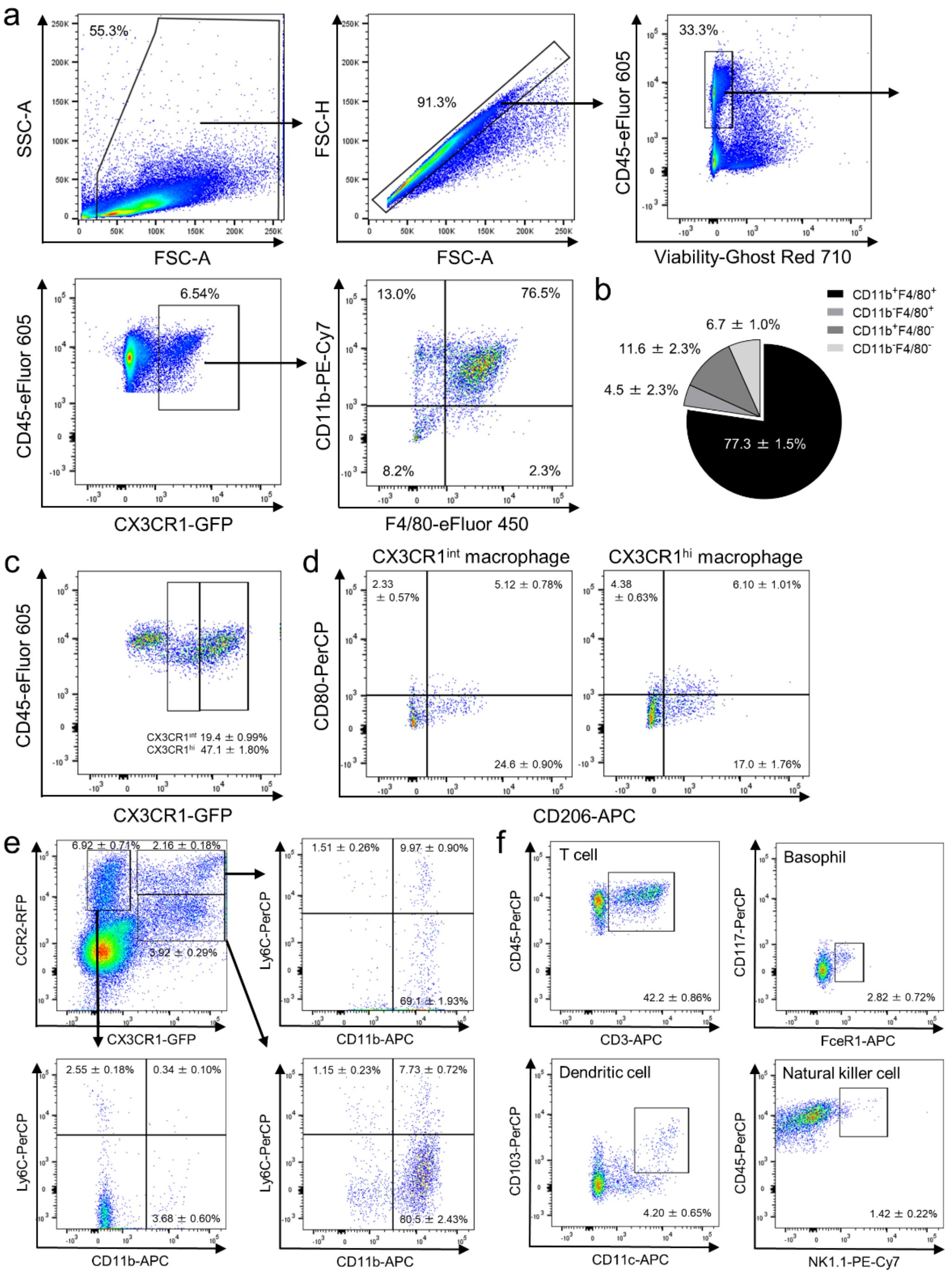

Supplementary Figure 1. Phenotype of CX3CR1$^+$ and CCR2$^+$ cell populations in lamina propria of the colon under steady state. (a) Flow cytometry sorting strategy for colonic lamina propria (LP) resident CX3CR1$^+$ cells. Results are representative of 2 independent experiments. (b) Quantification in proportion of CD11b$^+$F4/80$^+$ cells per CX3CR1$^+$ cells. Cells were pregated on size, viability, and CD45$^+$. n = 5. (c) Quantification in proportion of colonic LP CX3CR1^{int} and CX3CR1^{hi} macrophages at steady state. Cells were pregated on size, viability, CD45$^+$, CD103$^-$, CD11b$^+$ and F4/80$^+$. n = 8. (d) Macrophage phenotype (CD80$^+$CD206$^-$ or CD80$^-$CD206$^+$) in CX3CR1^{int} and CX3CR1^{hi} macrophages at steady state. Cells were pregated on size, viability, CD45$^+$, CD103$^-$, CD11b$^+$, F4/80$^+$ and CX3CR1$^{int/hi}$. n = 4. (e) Flow cytometry analysis of CD11b and Ly6C positivity in CX3CR1$^-$CCR2$^+$, CX3CR1$^+$CCR2^{hi}, and CX3CR1$^+$CCR2^{lo} cell population obtained from colonic LP under steady state. Cells were pregated on size, viability, and CD45$^+$. n = 8. (f) Phenotype of CD45$^+$CX3CR1$^-$CCR2$^+$ cells in colonic LP. n = 5. Data represent mean ± SEM. Source data are provided as a Source Data file.

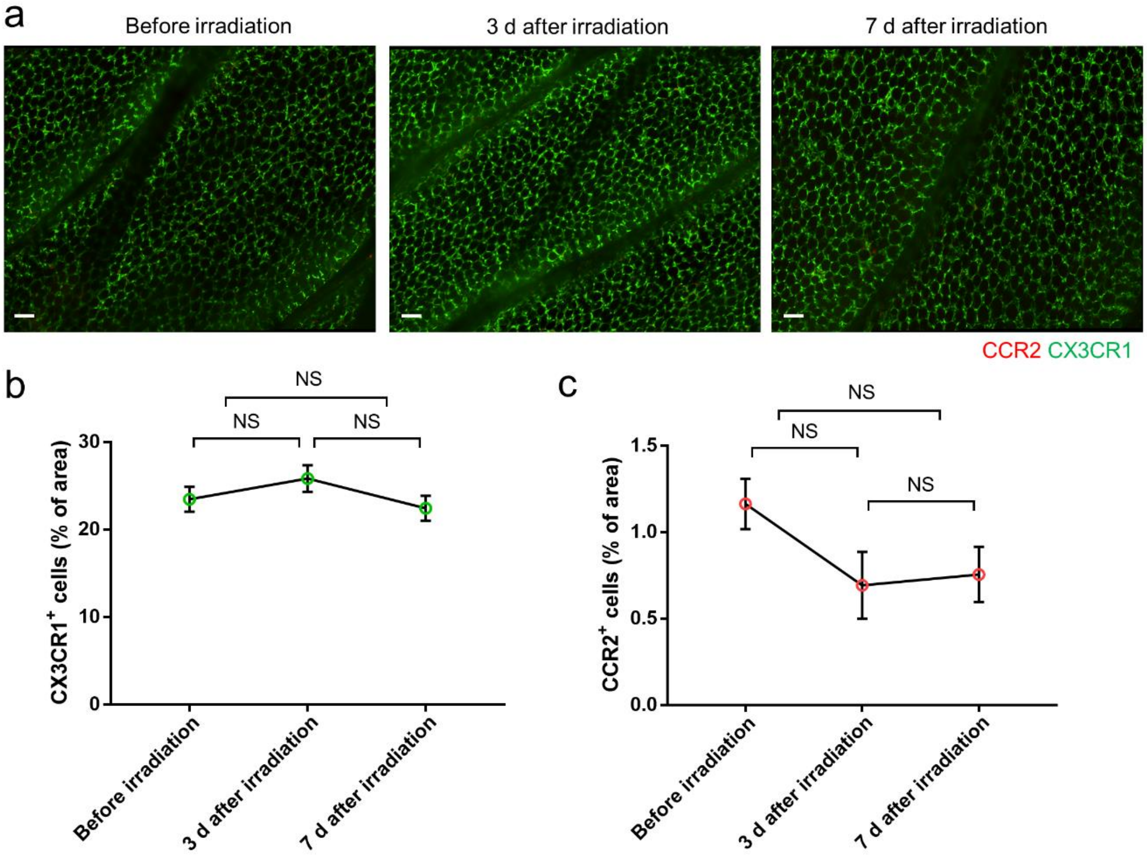

Supplementary Figure 2. Irradiation does not affect the resident population of intestinal CX3CR1⁺ macrophages. (a) Representative stitch images of colonic LP in *Cx3cr1*$^{GFP/+}$*Ccr2*$^{RFP/+}$ mice before irradiation and at 3 or 7 days after irradiation. Quantification of (b) $CX3CR1^+$ cells and (C) $CCR2^+$ cells (% of area). n = 3-6 at each time point. Data represent mean ± SEM. NS, not significant. Source data are provided as a Source Data file.

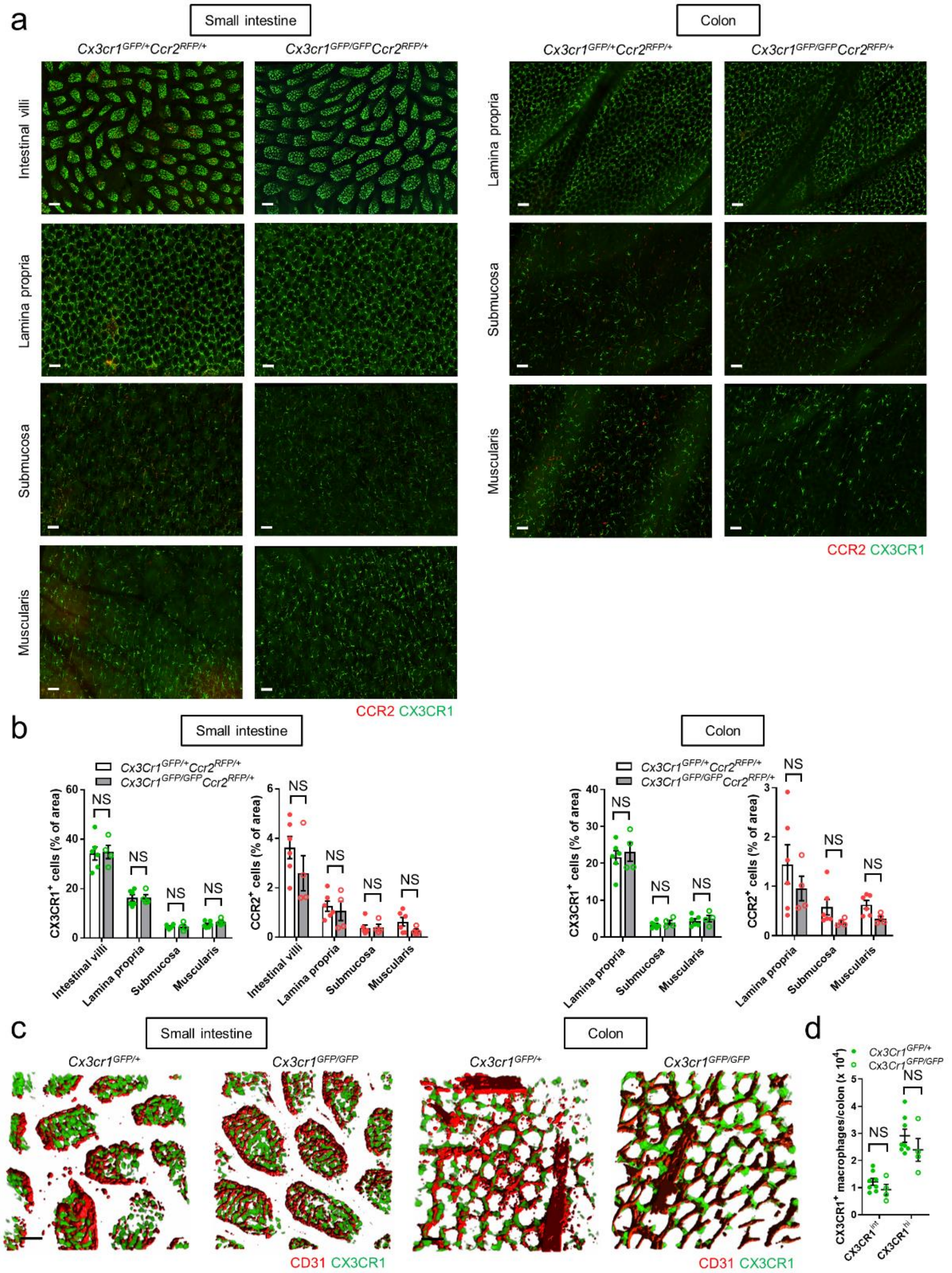

Supplementary Figure 3. CX3CR1 deficiency does not affect the distribution and morphology of intestinal CX3CR1^{+} macrophages. (a) Representative stitch images (from 12 different field of view) of small intestinal villi, lamina propria, submucosa and muscularis in *Cx3cr1*$^{GFP/+}$*Ccr2*$^{RFP/+}$ and *Cx3cr1*$^{GFP/GFP}$*Ccr2*$^{RFP/+}$ (CX3CR1 deficient) mice. Scale bars, 100 μm. (b) Quantification of CX3CR1^{+} cells or CCR2^{+} cells per field of view in each layer of the intestine in *Cx3cr1*$^{GFP/+}$*Ccr2*$^{RFP/+}$ and *Cx3cr1*$^{GFP/GFP}$*Ccr2*$^{RFP/+}$ mice. The area where the intestinal tissue does not exist was excluded from the calculation. n = 4-6 per group. (c) Representative 3D reconstructed images of CX3CR1^{+} macrophages in small intestinal villi and colonic lamina propria. (d) Quantification of the number of CX3CR1^{int} and CX3CR1^{hi} macrophages by flow cytometry in *Cx3cr1*$^{GFP/+}$ and *Cx3cr1*$^{GFP/GFP}$ mice. Cells were gated on size, viability, CD45^{+}, CD103^{-}, CD11b^{+}, F4/80^{+} and CX3CR1$^{int/hi}$. n = 4-8 per group. Data represent mean ± SEM. NS, not significant. Source data are provided as a Source Data file.

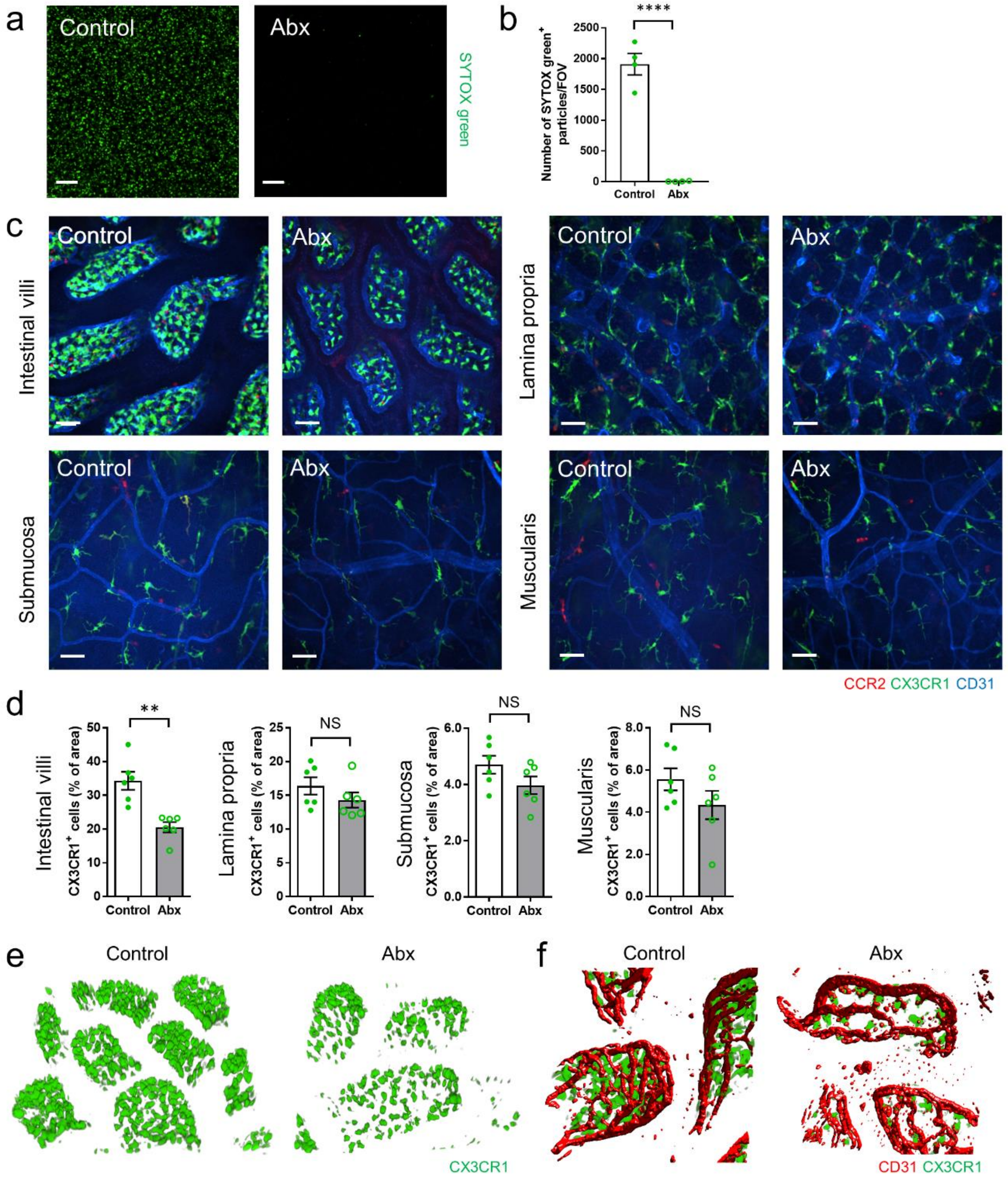

Supplementary Figure 4. Gut microbiota affects the distribution of small intestinal CX3CR1⁺ macrophages. (a) Representative images of SYTOX green⁺ particles in intestinal contents of SPF control and Abx-treated mice. Scale bars, 50 µm. (b) Quantification of the number of SYTOX green⁺ particle per field of view. n = 4 per group. (c) Representative images of small intestinal villi, lamina propria, submucosa and muscularis in *Cx3cr1*$^{GFP/+}$*Ccr2*$^{RFP/+}$ mice (The left column is SPF control, the right column is Abx-treated mice). Scale bars, 50 µm. (d) Quantification of CX3CR1⁺ cells per field of view in each layer of the small intestine in control and Abx-treated mice. The area where the intestinal tissue does not exist was excluded from the calculation. n = 6 per group. (e), (f) Representative 3D reconstructed images of CX3CR1⁺ macrophages in small intestinal villi in control and Abx-treated mice. Data are representative of five independent experiments. Data represent mean ± SEM. *$p < 0.05$, **$p < 0.01$, ****$p < 0.0001$, NS, not significant. Source data are provided as a Source Data file.

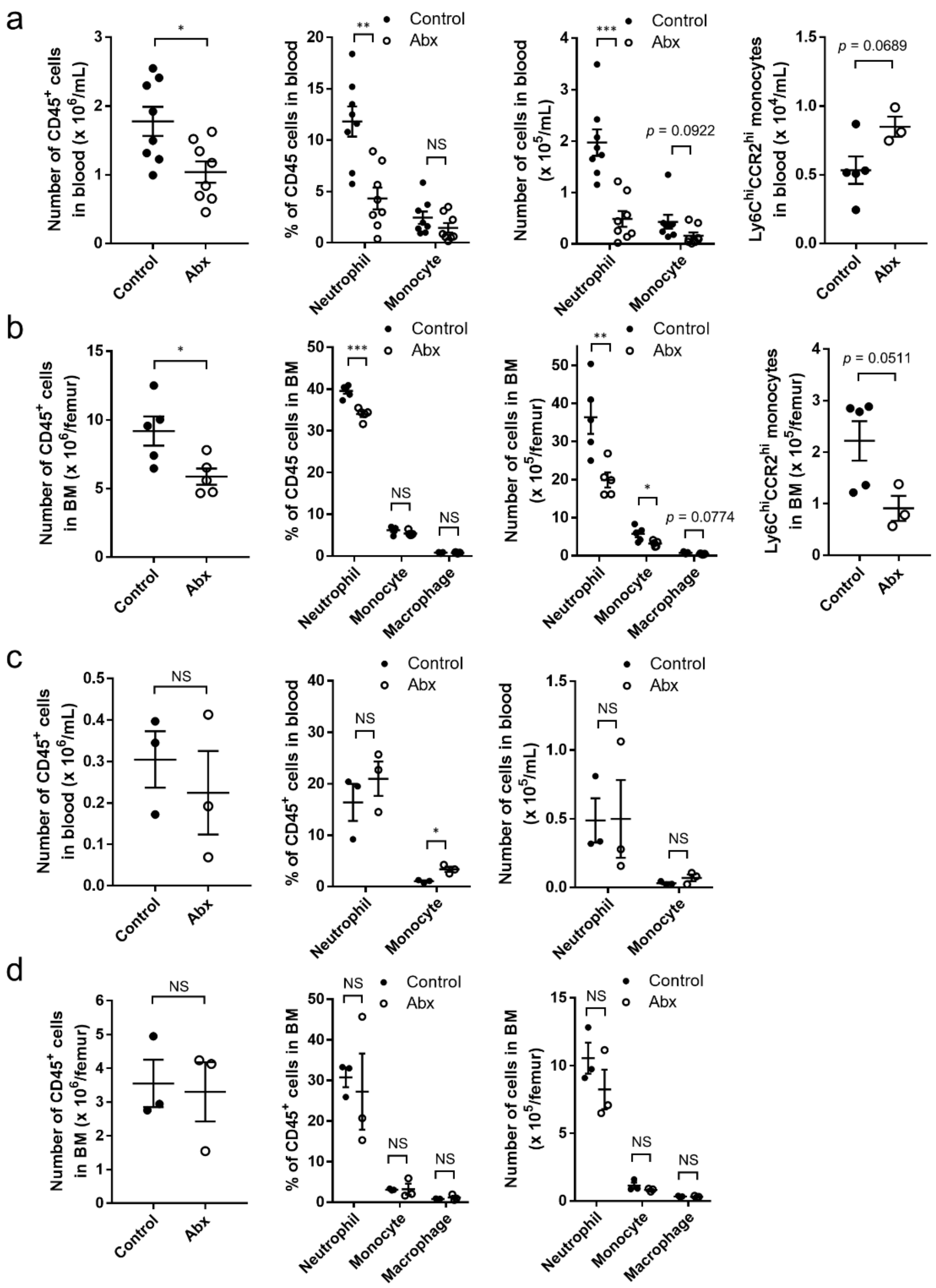

Supplementary Figure 5. Gut microbiota affects homeostasis of innate immune cells in blood and bone marrow. (a) Total number of leukocytes and proportion/number of innate immune cell subsets (neutrophil; gated on size, viability, $CD45^+$, $CD11b^+$, $Ly6G^+$, monocyte; gated on size, viability, $CD45^+$, $CD11b^+$, $Ly6G^{low}$, $Ly6C^+$, $Ly6C^{hi}CCR2^{hi}$ monocyte; pregated on size, viability, $CD45^+$, $CD11b^+$) in blood of control and Abx-treated mice at steady state. n = 3-8 per group. (b) Total number of leukocytes and proportion/number of innate immune cell subsets (neutrophil, monocyte, macrophage; gated on size, viability, $CD45^+$, $CD11b^+$, $F4/80^+$, $Ly6C^{hi}CCR2^{hi}$ monocyte) in bone marrow of control and Abx-treated mice at steady state. n = 3-5 per group. (c) Total number of leukocytes and proportion/number of innate immune cell subsets (neutrophil, monocyte) in blood of control and Abx-treated mice at 2 weeks after bone marrow transplantation (BMT). n = 3 per group. (d) Total number of leukocytes and proportion/number of innate immune cell subsets (neutrophil, monocyte, macrophage) in bone marrow of control and Abx-treated mice at 2 weeks after BMT. n = 3 per group. Data represent mean ± SEM. *$p < 0.05$, **$p < 0.01$, ***$p < 0.001$, NS, not significant. Source data are provided as a Source Data file.

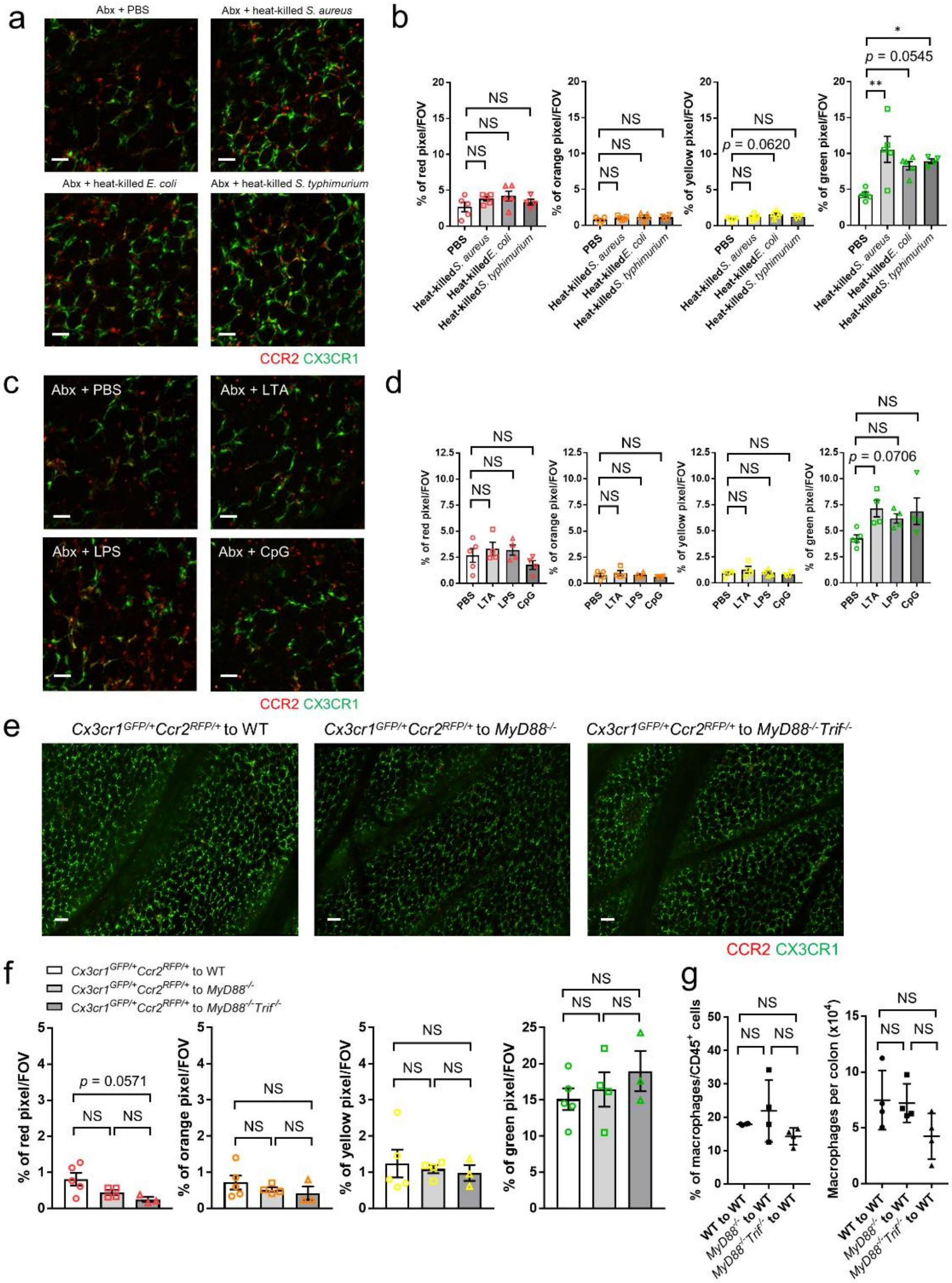

Supplementary Figure 6. TLRs and MyD88 or MyD88/Trif signaling are not key pathways in turnover of intestinal macrophages in response to bacterial products. (a) Representative images in colonic LP of Abx-treated mice who underwent BMT from *Cx3cr1*$^{GFP/+}$*Ccr2*$^{RFP/+}$ mice. Mice were treated by PBS, or heat-killed bacteria including *S. aureus*, *E. coli*, or *S. typhimurium* for 6 weeks after BMT. Scale bars, 50 μm. (b) Quantification of monocyte hues 6 weeks after BMT in each group. n = 4-5 per group. (c) Representative images of colonic lamina propria in Abx-treated C57BL/6 mice at 6 weeks after BMT from *Cx3cr1*$^{GFP/+}$*Ccr2*$^{RFP/+}$ mice. Mice were treated by oral administration of PBS, LTA, LPS, or CpG. Scale bars, 50 μm. (d) Quantification of monocyte hues 6 weeks after BMT in each group. n = 4-5 per group. (e) Representative stitch images of colonic lamina propria and (f) quantification of monocyte hues in WT, *MyD88*$^{-/-}$, or *MyD88*$^{-/-}$*Trif*$^{-/-}$ mice at 6 weeks after BMT from *Cx3cr1*$^{GFP/+}$*Ccr2*$^{RFP/+}$ mice. Scale bars, 100 μm. n = 3-5 per group. (g) Proportion and number of intestinal macrophages in WT mice 6 weeks after BMT from WT, *MyD88*$^{-/-}$, or *MyD88*$^{-/-}$*Trif*$^{-/-}$ mice. Cells were pregated on size, viability, CD45^{+}, CD11b^{+}, and F4/80^{+}. n = 4 per group. Data represent mean ± SEM. *$p < 0.05$, **$p < 0.01$, NS, not significant. Source data are provided as a Source Data file.

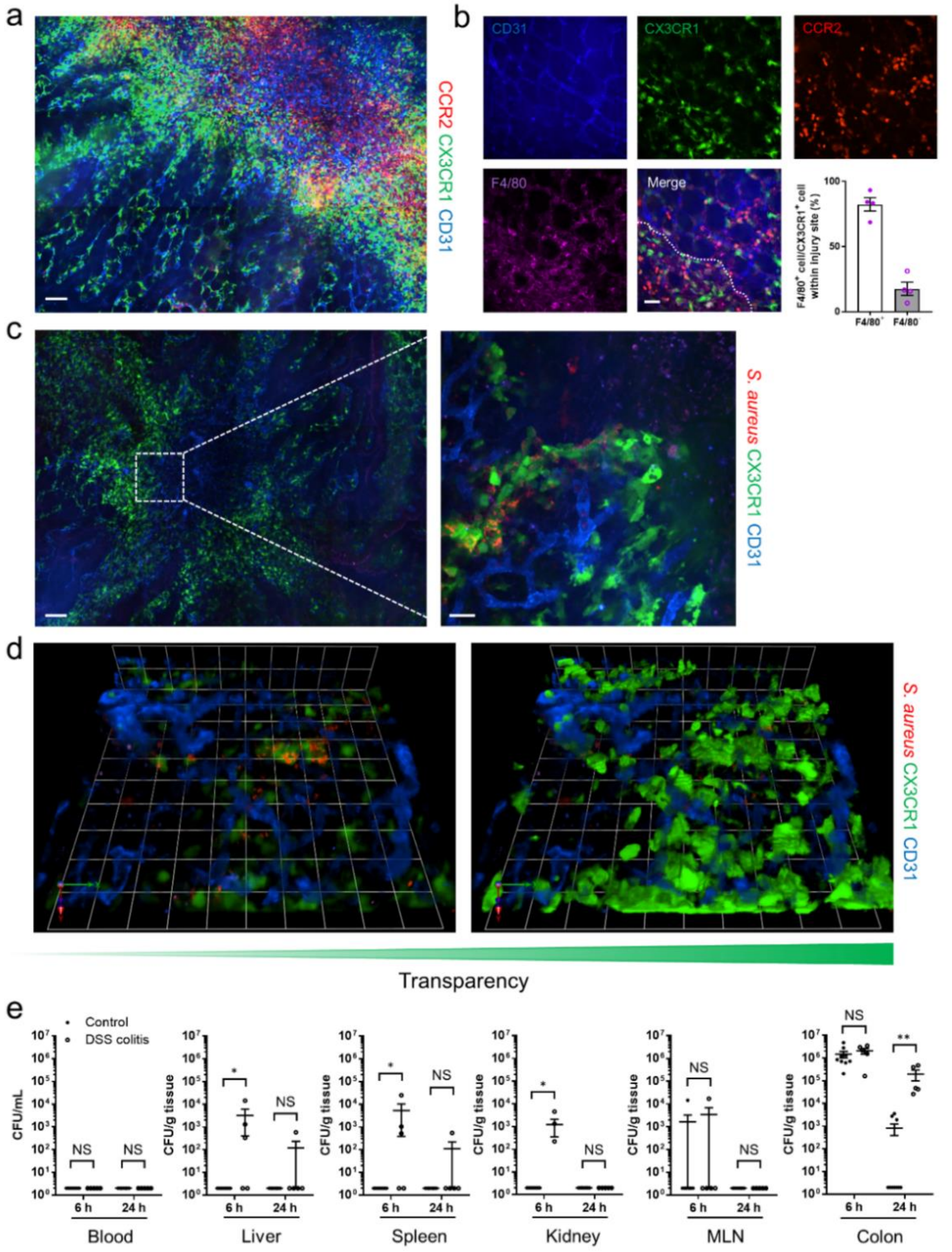

Supplementary Figure 7. CX3CR1^{+} macrophages catch *Staphylococcus aureus* that invade the damaged area induced by DSS colitis. (a) Representative stitch image of colonic lamina propria in *Cx3cr1$^{GFP/+}$Ccr2$^{RFP/+}$* mice 5 days after the start of 4% DSS. Scale bar, 100 μm. (b) Representative images of F4/80^{hi} macrophages (purple) at the site of intestinal injury in *Cx3cr1$^{GFP/+}$Ccr2$^{RFP/+}$* mice under acute DSS colitis by topical application of F4/80 antibody. Intestinal vasculature was visualized by anti-CD31 (blue) antibody. White dotted line indicates injury area. Scale bar, 50 μm. The proportion of F4/80^{+} cells/CX3CR1^{+} cells was quantified. n = 4. (c) Representative stitch image of colonic lamina propria in *Cx3cr1$^{GFP/+}$* mice 6 hrs after mCherry MW2 gavage under acute DSS colitis. Higher magnification of the indicated area is shown in right. Scale bar, 100 μm (left) and 20 μm (right). (d) 3D images of mCherry MW2 phagocytosed by CX3CR1^{+} macrophages. 1 Unit = 23.3 μm. Transparency adjustment of CX3CR1 (green) was applied (left) to allow for visualization of *S. aureus* inside CX3CR1^{+} macrophages. (e) CFU counts at 6 and 24 hrs after *S. aureus* gavage in control and acute DSS colitis mice. n = 5-10 per group. Data represent mean ± SEM. *$p < 0.05$, **$p < 0.01$, NS, not significant. Source data are provided as a Source Data file.

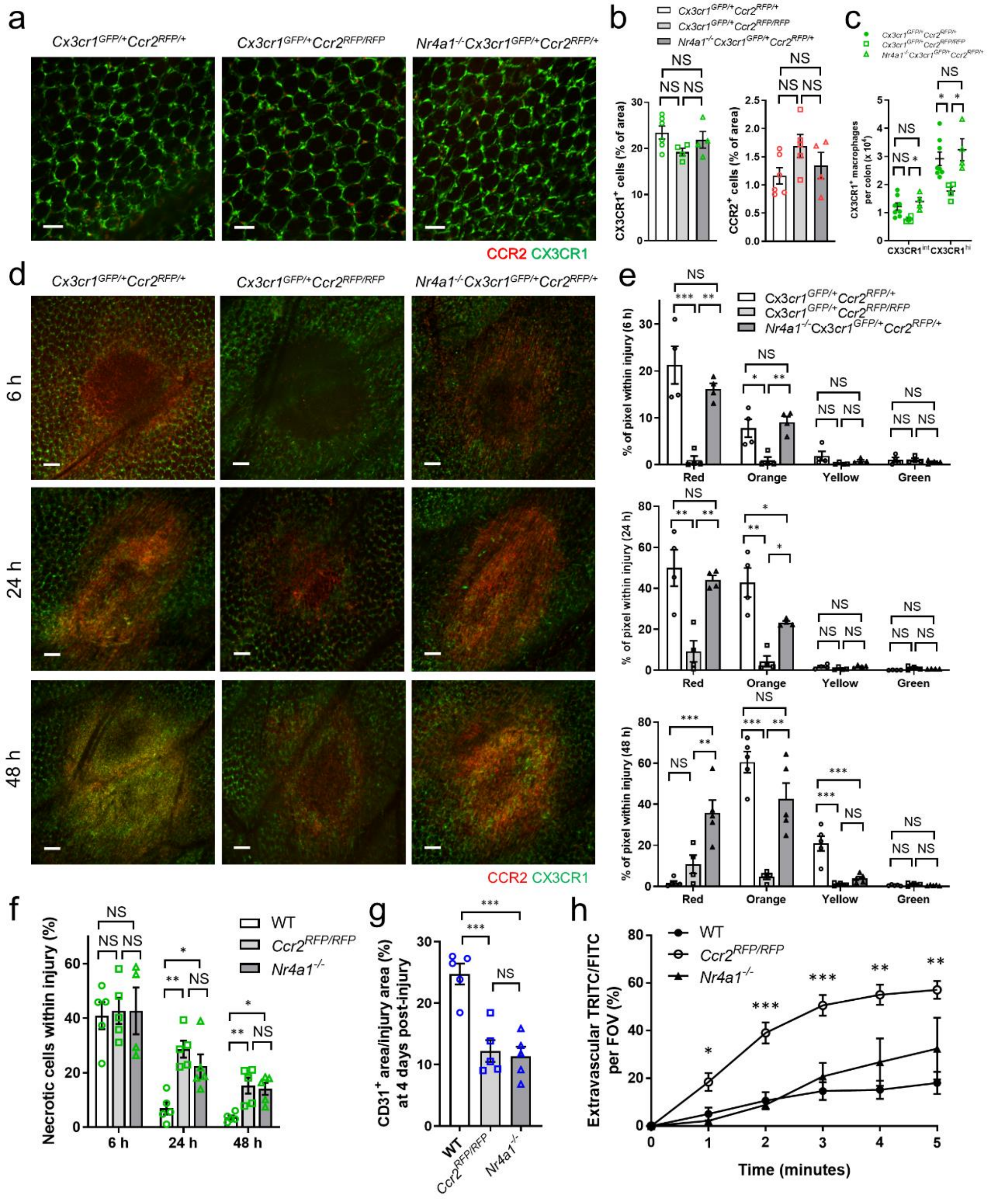

Supplementary Figure 8. Accumulation of reparative CX3CR1^{+} monocytes at the site of sterile injury is dependent on the CCR2 receptor and transcription factor Nr4a1. (a) Representative images of colonic LP in *Cx3cr1$^{GFP/+}$Ccr2$^{RFP/+}$*, *Cx3cr1$^{GFP/+}$Ccr2$^{RFP/RFP}$*, and *Nr4a1$^{-/-}$Cx3cr1$^{GFP/+}$Ccr2$^{RFP/+}$* mice. Scale bars, 50 µm. (b) Quantification of the area in CX3CR1^{+} cells or CCR2^{+} cells per field of view. n = 4-6 per group. (c) Quantification of the number of CX3CR1^{int} and CX3CR1^{hi} macrophages by flow cytometry in each group. Cells were gated on size, viability, CD45^{+}, CD103^{-}, CD11b^{+}, F4/80^{+} and CX3CR1$^{int/hi}$. n = 4-8 per group. (d) Representative images taken from 6 to 48 hrs after focal intestinal injury and (e) quantification of their monocyte hues within injury in *Cx3cr1$^{GFP/+}$Ccr2$^{RFP/+}$*, *Cx3cr1$^{GFP/+}$Ccr2$^{RFP/RFP}$*, and *Nr4a1$^{-/-}$Cx3cr1$^{GFP/+}$Ccr2$^{RFP/+}$* mice. Scale bars, 100 µm. n = 4-6 per group. Quantification of (f) SYTOX green^{+} and (g) CD31^{+} area within injury at indicated time points. n = 4-5 per group. (h) Quantification of extravascular TRITC/FITC^{+} area/FOV. n = 3-5 per group. Data represent mean ± SEM. *$p < 0.05$, **$p < 0.01$, ***$p < 0.001$, NS, not significant. Source data are provided as a Source Data file.
