# Supplementary material for: Perivascular localization of macrophages in the intestinal mucosa is regulated by Nr4a1 and the microbiome
Source: Nat Commun. 2020 Mar 12;11:1329. doi: 10.1038/s41467-020-15068-4 (PMC7067862; doi:10.1038/s41467-020-15068-4)
Supplement: Supplementary file 6 — Reporting Summary [file 41467_2020_15068_MOESM6_ESM.pdf]

nature research

Corresponding author(s): Paul Kubes

Last updated by author(s): Feb 8, 2020

Reporting Summary

Nature Research wishes to improve the reproducibility of the work that we publish. This form provides structure for consistency and transparency in reporting. For further information on Nature Research policies, see Authors & Referees and the Editorial Policy Checklist.

Statistics

For all statistical analyses, confirm that the following items are present in the figure legend, table legend, main text, or Methods section.

n/a	Confirmed	
☐	☒	The exact sample size (n) for each experimental group/condition, given as a discrete number and unit of measurement
☐	☒	A statement on whether measurements were taken from distinct samples or whether the same sample was measured repeatedly
☐	☒	The statistical test(s) used AND whether they are one- or two-sided<br>*Only common tests should be described solely by name; describe more complex techniques in the Methods section.*
☐	☒	A description of all covariates tested
☐	☒	A description of any assumptions or corrections, such as tests of normality and adjustment for multiple comparisons
☐	☒	A full description of the statistical parameters including central tendency (e.g. means) or other basic estimates (e.g. regression coefficient) AND variation (e.g. standard deviation) or associated estimates of uncertainty (e.g. confidence intervals)
☒	☐	For null hypothesis testing, the test statistic (e.g. F, t, r) with confidence intervals, effect sizes, degrees of freedom and P value noted<br>*Give P values as exact values whenever suitable.*
☒	☐	For Bayesian analysis, information on the choice of priors and Markov chain Monte Carlo settings
☒	☐	For hierarchical and complex designs, identification of the appropriate level for tests and full reporting of outcomes
☒	☐	Estimates of effect sizes (e.g. Cohen's d, Pearson's r), indicating how they were calculated

Our web collection on statistics for biologists contains articles on many of the points above.

Software and code

Policy information about availability of computer code

Data collection	Volocity software 6.1 (PerkinElmer) was used to drive the confocal microscope and for 3D rendering, acquisition, and analysis of images.
Data analysis	Flow cytometry data were analyzed using FlowJo v10 software (Tree Star). Imaging data were analyzed using Image J v1.45 software package (NIH). Cytokine data were obtained using Luminex assay and analyzed with StarStation V.2.3 (Applied Cytometry System). Statistical analyses were performed using GraphPad Prism v7.0 software (GraphPad Software Inc.).

For manuscripts utilizing custom algorithms or software that are central to the research but not yet described in published literature, software must be made available to editors/reviewers. We strongly encourage code deposition in a community repository (e.g. GitHub). See the Nature Research guidelines for submitting code & software for further information.

Data

Policy information about availability of data

All manuscripts must include a data availability statement. This statement should provide the following information, where applicable:

- Accession codes, unique identifiers, or web links for publicly available datasets
- A list of figures that have associated raw data
- A description of any restrictions on data availability

The authors declare that the data supporting the findings of this study are available within the paper and its supplementary information files.

Field-specific reporting

Please select the one below that is the best fit for your research. If you are not sure, read the appropriate sections before making your selection.

☒ Life sciences ☐ Behavioural & social sciences ☐ Ecological, evolutionary & environmental sciences

For a reference copy of the document with all sections, see nature.com/documents/nr-reporting-summary-flat.pdf

Life sciences study design

All studies must disclose on these points even when the disclosure is negative.

Sample size	Sample sizes were chosen based on previous experiments conducted in our laboratories.
Data exclusions	No data were excluded.
Replication	Experiments were reliably reproduced. Experimental findings were reproduced at least twice.
Randomization	Mice of the same age/sex were randomly allocated to each experimental groups.
Blinding	Investigators were not blinded.

Reporting for specific materials, systems and methods

We require information from authors about some types of materials, experimental systems and methods used in many studies. Here, indicate whether each material, system or method listed is relevant to your study. If you are not sure if a list item applies to your research, read the appropriate section before selecting a response.

Materials & experimental systems

n/a	Involved in the study	
☐	☒	Antibodies
☒	☐	Eukaryotic cell lines
☒	☐	Palaeontology
☐	☒	Animals and other organisms
☒	☐	Human research participants
☒	☐	Clinical data

Methods

n/a	Involved in the study	
☒	☐	ChIP-seq
☐	☒	Flow cytometry
☒	☐	MRI-based neuroimaging

Antibodies

Antibodies used

Anti-CD3 (clone 17A2) eBioscience
Anti-CD11b (clone M1/70) eBioscience
Anti-CD11c (clone N418) eBioscience
Anti-CD31 (clone 390) eBioscience
Anti-CD45 (clone 30-F11) eBioscience
Anti-FceR1 (clone MAR-1) eBioscience
Anti-F4/80 (clone BM8) eBioscience
Anti-CD80 (clone 16-10A1) Biolegend
Anti-CD117 (clone 2B8) Biolegend
Anti-CD206 (clone C068C2) Biolegend
Anti-CX3CR1 (SA011F11) Biolegend
Anti-Ly6C (clone HK1.4) Biolegend
Anti-Ly6G (clone 1A8) Biolegend
Anti-NK1.1 (clone PK136) BD Pharmingen
Anti-CD103 (clone M290) BD Pharmingen
Anti-CD16/32 (clone 2.4G2) Bio X Cell

Validation

The dose of antibody used in this study was determined based on previous experiments conducted in our laboratories.

Animals and other organisms

Policy information about studies involving animals; ARRIVE guidelines recommended for reporting animal research

Laboratory animals

C57BL/6 mice, Cx3cr1GFP/+ (knock-in), Cx3cr1GFP/GFP (CX3CR1-deficient), and Nr4a1-/- mice were obtained from The Jackson Laboratory. Generation of Ccr2RFP/RFP (CCR2-deficient) and Ccr2RFP/+ (knock-in) mice have been previously described.

Cx3cr1GFP/+Ccr2RFP/+ mice were generated by crossing Cx3cr1GFP/GFPCcr2RFP/RFP mice with C57BL/6 mice. Nr4a1-/-Cx3cr1GFP/+Ccr2RFP/+ mice were generated by crossing Nr4a1-/-Cx3cr1GFP/GFPCcr2RFP/RFP mice with Nr4a1-/- mice. Myd88-/- and Myd88-/-Trif-/- double knockout mice were a gift from Dr. S. Akira (Osaka University, Japan). Csf1rLsL-DTR mice were a gift from Dr. M. Nussenzweig. To deplete intestinal macrophages, Csf1rLsL-DTR mice were crossed LysMcre mice and DT (4 ng/g mouse body weight) was administered intraperitoneally 24 before the experiments. Germ free Cx3cr1GFP/GFP mice were bred and maintained in flexible film isolators at the IMC, University of Calgary. Mice of 8-12 weeks of age were used for experiments.

Wild animals	The study does not involve wild animals.
Field-collected samples	The study does not involve samples collected from the field.
Ethics oversight	All experiments were approved by the University of Calgary Animal Care Committee and were in compliance with guidelines established by the Canadian Council for Animal Care.

Note that full information on the approval of the study protocol must also be provided in the manuscript.

Flow Cytometry

Plots

Confirm that:

☒ The axis labels state the marker and fluorochrome used (e.g. CD4-FITC).

☒ The axis scales are clearly visible. Include numbers along axes only for bottom left plot of group (a 'group' is an analysis of identical markers).

☒ All plots are contour plots with outliers or pseudocolor plots.

☒ A numerical value for number of cells or percentage (with statistics) is provided.

Methodology

Sample preparation	Cells were isolated from blood, femur and colon. Residual red blood cells were lysed using ACK lysing buffer (Invitrogen). Single-cell suspensions of bone marrow were generated by mechanical disruption through a 40-µm nylon mesh (BD Bioscience). Colonic lamina propria cells were isolated as described in methods. The cells were blocked using anti-CD16/32 antibody (2.4G2 clone; Bio X Cell) for 30 min. Then, cells were stained for 30 min with antibodies for specified markers.
Instrument	FACSCanto and LSR-II (BD Biosciences)
Software	FlowJo v10 software (Tree Star)
Cell population abundance	Sorting was not performed.
Gating strategy	Cell debris were first excluded by SSC-A and FSC-A. Then, doublets were excluded using single-cell gating based on FSC-H and FSC-A. CD45 and viability dye staining were used to gate the live leukocytes. Further gating strategies depend on the experimental setup.

☒ Tick this box to confirm that a figure exemplifying the gating strategy is provided in the Supplementary Information.
